# Resilience, and positive parenting in parents of children with syndromic autism and intellectual disability. Evidence from the impact of the COVID‐19 pandemic on family's quality of life and parent–child relationships

**DOI:** 10.1002/aur.2825

**Published:** 2022-10-04

**Authors:** Corneliu Bolbocean, Kayla B. Rhidenour, Maria McCormack, Bernhard Suter, Jimmy Lloyd Holder

**Affiliations:** ^1^ The Nuffield Department of Primary Care Health Sciences University of Oxford Oxford UK; ^2^ Department of Communication Baylor University Waco Texas USA; ^3^ Jan and Dan Duncan Neurological Research Institute Texas Children's Hospital Houston Texas USA; ^4^ Division of Neurology and Developmental Neuroscience, Department of Pediatrics Baylor College of Medicine Houston Texas USA

**Keywords:** autism, COVID‐19, families of autistic children, intellectual disabilities, parents, Phelan–McDermid syndrome, resilience, Rett syndrome, SYNGAP1‐ID

## Abstract

Family quality of life (FQoL) outcomes collected during the first year of COVID‐19 has been combined with 2018 data to estimate the outbreak's impact on parental outcomes on a sample of 230 families with syndromic autistic children and those with intellectual disabilities (IDs). Despite challenges imposed by the COVID‐19 outbreak, our study found that FQoL outcomes reported by participating parents during the first year of COVID‐19 appears to be similar to ratings from a prepandemic study of families with the same conditions. Parents of children in our sample generally displayed a stable functioning trajectory as measured by the validated FQoL instrument. Across syndromic autistic groups considered, families reported that their relationships with their children were positive. Our findings provide evidence of families' resilience which might explain the presence of positive parent–child interactions during COVID‐19. Exploring mechanisms which would explain how families with autistic and ID children confront, manage disruptive experiences, and buffer COVID‐19 induced stress is a fruitful direction for future research.

## Lay Summary

Despite challenges imposed by the COVID‐19 outbreak, our study found that the quality of life reported by participating families during the first year of COVID‐19 appears to be similar to ratings from a prepandemic study of families with the same conditions. The COVID‐19 pandemic and related lockdowns were found to be positively associated with parent–child bonding, relational satisfaction, and increased emotional connection between parents and their children. Our findings provide evidence of families' resilience which might explain the presence of positive parental–child interactions during COVID‐19.

## INTRODUCTION

The COVID‐19 pandemic has had an undisputed impact on children and families. Recent research has found an increased risk of negative mental health, neurodevelopmental, and health‐related quality of life outcomes among children (Courtenay & Perera, 2020; Ehrler et al., 2021; Masi et al., 2021; Summers et al., 2021) and individuals diagnosed with neurodevelopmental disabilities such as intellectual disability (ID) or with autism spectrum disorders (ASD) (Bolbocean et al., 2022; den Houting, 2020; Eshraghi et al., 2020; Pellicano & Stears, 2020; Thompson & Nygren, 2020; Trip et al., 2022). The challenges of caring for a child with a developmental disability are well documented in the literature. Research has shown that the COVID‐19 pandemic has increased the likelihood of factors known to be associated with parental distress and quality of life outcomes (Hsiao et al., 2017; McStay et al., 2014; Vasilopoulou & Nisbet, 2016) and families of autistic children or children with IDs experienced an increase of intensity and frequency of behavioral problems with their children (Colizzi et al., 2020; Morris et al., 2021). Furthermore, children's characteristics such as behavioral phenotype, severity of the impairment, as well as age, and gender were also associated with parental stress and the overall emotional well‐being of parents (Alhuzimi, 2021).

During the first wave of COVID‐19, governments undertook comprehensive measures to contain the spread and effects of the virus in their respective countries with the majority of nonessential businesses, healthcare, and social care services stopped (Haug et al., 2020; Prem et al., 2020). As the pandemic progressed over time, a distinct shift occurred during the autumn of 2020, with countries around the globe relaxing their previously implemented social restrictions to deter the spread of COVID‐19 (Lordan et al., 2020; Sheikh et al., 2020). We point to this systematic easing of restrictions and loosening of lockdown policies which allowed for children to return to schools and other educational facilities as a particularly interesting moment in time for expanding our existing knowledge of the impacts COVID‐19 had on parents and their children. There is little evidence regarding the impact of easing restrictions during autumn 2020 on family quality of life (FQoL) of caregivers of children with developmental disabilities.

Furthermore, while recent research has generally highlighted the negative impacts of the COVID‐19 pandemic on autistic individuals, the literature overall highlights contrasting evidence around the effects of the pandemic on family caregivers and their interactions with children (Bailey et al., 2021; Fontanesi et al., 2020; Greenway & Eaton‐Thomas, 2020; Mumbardó‐Adam et al., 2021; Narzisi, 2020; Navas et al., 2022; O'Connor Bones et al., 2022; Tsibidaki, 2021; Wanjagua et al., 2022). The evidence finds that parents of autistic children were very stressed and struggled during the early phase of the pandemic (Amorim et al., 2020; Asbury et al., 2021; Pellicano et al., 2021). At the same time, emergent research has also highlighted some positive impacts of the COVID‐19 pandemic on families with autistic children or children with IDs (Fontanesi et al., 2020; Greenway & Eaton‐Thomas, 2020; Mumbardó‐Adam et al., 2021).

The COVID‐19 crisis likely encouraged the activation of protective mechanisms within families such as psychosocial resilience. Resilience is understood as the ability of beating the odds under adverse conditions for families and their children, and has been linked to positive outcomes (Bitsika et al., 2013; Ekas & Rafferty, 2019; Ilias et al., 2018; Luthar et al., 2000; Ruiz‐Robledillo et al., 2014). Resilience is a protective factor that fosters the development of positive outcomes among individuals exposed to unfavorable life circumstances (Garmezy, 1991). In the care context, resilience is understood as the ability to recover from the stress of a caretaking role as well as the capacity to promote a successful adaptation and acceptance of one's care situation (Lin et al., 2013). Thus, research reports that families of children with special educational needs and disabilities were able to activate mechanisms of self‐efficacy and meaning into their lives increasing resilience and maintain their balance and mental health (Tsibidaki, 2021). These families were able to do this despite experiencing difficult situations and anxiety due to the pandemic.

The quality of parent–child (PC) relationships has been found to be a significant predictor of outcomes for children especially during periods of stress and uncertainty (Kerns et al., 2014; Kiliç et al., 2011; Masten & Narayan, 2012). Specifically, constructive communication and conflict resolution are known psychosocial facets of relational resilience (Walsh, 1996). Bonding and quality time have been found to illustrate family resilience during COVID‐19, and each were associated with improved security for children during times of stress (Prime et al., 2020). Overall, the resilience related constructs assessed have been shown to either directly impact or mediate processes linking stressful events to quality or stability of family relationships (Prime et al., 2020; Randall & Bodenmann, 2017; Walsh, 1996).

Studies based on cross sectional designs collected during early months of COVID‐19 reported that families with autistic children registered greater behavioral problems and parental distress compared with families of nonautistic children during the COVID‐19 restrictions (Levante et al., 2021). However, the overall evidence regarding the impact of the pandemic on outcomes in families with autistic children or children with IDs remains inconclusive (Bailey et al., 2021; Fontanesi et al., 2020; Greenway & Eaton‐Thomas, 2020; Mumbardó‐Adam et al., 2021; Narzisi, 2020; Navas et al., 2022; O'Connor Bones et al., 2022; Pellicano et al., 2021; Tsibidaki, 2021; Wanjagua et al., 2022). One explanation is that much of the research into the impact of the pandemic on parental outcomes is subject to methodological challenges. Specifically, the main methodological concern of many existing studies is the lack of repeated observations collected before and during the pandemic that would allow rigorous longitudinal research designs to ascertain any potential impacts of COVID‐19 on caregivers' outcomes. Furthermore, although resilience has been identified as a protective factor in the stress adaptation process for autistic families (Kotera et al., 2021), to our knowledge no studies have analyzed these positive effects in families impacted by syndromic autism or ID during the pandemic. As a result, reliable data to inform a comprehensive discussion regarding the overall impact of COVID‐19 on families with autistic children or children with IDs are limited. Moreover, there is no research which would ascertain the impact of the pandemic on FQoL or on PC relationships among syndromic autistic and ID children which are at risk of adverse outcomes.

To overcome the limitations associated with analyses restricted to cross sectional designs or cohorts, the use of FQoL data consolidated before and during the pandemic is advantageous because it allows for detailed examination of the impact of COVID‐19 on caregivers' outcomes across different time points. Such research should provide much needed data to inform policy debates and efforts around families of children with autism, ID or with developmental delays. In this study, we focused on families with children diagnosed with Phelan–McDermid syndrome (PMD), Rett syndrome (RTT), and *SYNGAP1*‐related ID (*SYNGAP1*‐ID). These disorders strongly predispose a child to a diagnosis of autism or ID (Berryer et al., 2013; De Rubeis et al., 2018; Holder Jr et al., 2019; Jimenez‐Gomez et al., 2019; Kaufmann et al., 2012; Neul, 2012; Oberman et al., 2015; Phelan et al., 2018; Vlaskamp et al., 2019) and these neurodevelopmental disorders display complex associations with autistic features and behavior problems consistent with autism (Holder Jr et al., 2019; Kaufmann et al., 2012; Phelan et al., 2018). Genetic syndromes with a high prevalence of autism are often referred to as syndromic autism because of their comorbid phenotypes of ID, epilepsy and dysmorphic features (Benvenuto et al., 2009; Sztainberg & Zoghbi, 2016). Moreover, these disorders have defined genetic etiologies, each potentially has a more homogeneous phenotype than idiopathic autism.^1^

Research had shown that the clinical traits of autistic individuals were associated with their wellbeing during the early phase of the pandemic (Alhuzimi, 2021; Colizzi et al., 2020). However, the evidence shows that PMD, RTT, and *SYNGAP1*‐ID, while they share common traits, have distinctive clinical characteristics. A previous study identified differences in FQoL average scores across these disorders related to family interaction, and parenting domains (Bolbocean et al., 2021). This study identified emotional well‐being as the most severely impacted measure among these same syndromic autisms with less impact on family interaction, parenting and disability‐related support. The heterogeneity among these disorders might potentially have a differential impact upon FQoL as a result of the COVID‐19 pandemic. However, previously reported FQoL differences across diagnoses did not adjust for the socioeconomic gradient of health. Thus, it is currently unknown whether, or to what degree, the socioeconomic factors might explain observed FQoL differences across PMD, RTT, or *SYNGAP1*‐ID. This is important to explore particularly during COVID‐19 in order to understand the possible mechanisms aimed at managing and buffering COVID‐19 induced disruptions within families with children with neurodevelopmental disabilities.

This study was designed to advance the following research aims: (a) to document parental COVID‐19 environments of children diagnosed with single‐gene disorders which are strong risk factors for developing an ASD, syndromic autism or ID: PMD, RTT, and *SYNGAP1*‐ID; (b) to assess the impact of COVID‐19 on FQoL outcomes using identical and validated measures collected between 2018 and during the first year of the pandemic on a sample of families recruited via identical methods; (c) to compare FQoL outcomes from earlier and later phases of the first year of the outbreak. Finally, (d) to evaluate the PC relationships and overall experiences of parenthood during the first year of COVID‐19, and to estimate the association between pandemic induced factors with those relationships and experiences.

## DATA

This study used data collected at two points in time: prior to COVID‐19, specifically during 2018 as previously described (Bolbocean et al., 2021), as well as during the first year of the COVID19 pandemic. Both studies recruited participating families using identical methods and sample frames. Between July 2020 and January 2021, 230 families with children diagnosed with PMD, RTT, and *SYNGAP1*‐ID completed self‐report questionnaires in order to ascertain COVID‐19 induced lifestyle changes, FQoL and PC relationships. Study participants were distributed as follows: 138 (60%) parents with children diagnosed with PMD, 50 (22%) parents of children diagnosed with *SYNGAP1*‐ID children and 42 (18%) parents of children diagnosed with RTT. This study was approved by Baylor College of Medicine's Institutional Review Board. A recruitment email provided access to an online Qualtrics survey distributed by: PMD Foundation, RettSyndrome.org and *SYNGAP1*‐ID Education and Research Foundation. In order to complete the web‐based questionnaire the participants had to read and understand English and have Internet access. Before completing the questionnaire, the respondents gave their informed consent.

### Parental environments during COVID‐19

We designed a novel survey to document the environments of caregivers of children diagnosed with a single‐gene disorder during the first year of the COVID‐19 pandemic. The following domains were assessed: overall health, behaviors, school activities, access to healthcare, daily routines, and household finances.

### Parent–child relationship during COVID‐19. COVID‐19 parent–child relationship scale

PC relationships and interactions were investigated using a set of questions designed by the study's authors and were informed by previously published research measures (Barnard & Kelly, 1990; Beurkens et al., 2013; Masse et al., 2007). The following resilience related constructs were ascertained: constructive communication, conflict resolution, and bonding. Constructive communication and conflict resolution have each been shown as keys to mediating processes that linked stressful events to overall relational stability (Karney & Bradbury, 1995; Walsh, 2015). However, bonding was found to be an important factor in overcoming crisis related challenges in families (Walsh, 1996).

To compute a comprehensive measure which assessed PC interactions, we designed an ad hoc P‐C scale. Based on the answers provided, we computed P‐C scales to evaluate the amount/frequency of positive parenting during the first year of COVID‐19 using a linear transformation of ordered answers into scores on a 0–100 scale, where 0 represents the worst possible outcome and 100 represents a perfect outcome. One hundred points were given for the most positive PC answer and zero points for the worst. For example, for the question “how often do you spend fun time with your child?” options were rated as follows: Always/Extremely = 100, Often/Very much = 75, Sometimes = 50, Seldom/Not too much = 25, Never/Hardly at all = 0. Additionally, for the question “how often do you criticize your child?” Always/Extremely = 0, Often/Very much = 25, Sometimes = 50, Seldom/Not too much = 75, Never/Hardly at all = 100.

### Comparison of assessment of FQoL scores collected before and during the COVID‐19 pandemic

We measured the quality of life outcomes of caregivers using the Beach Center family quality of life Scale. FQoL is a validated instrument designed to measure the quality of life of families with children (Perry & Isaacs, 2015; Rivard et al., 2017). The Beach Center FQoL Scale was administered as a 25‐item inventory utilizing satisfaction as the primary response format with five dimensions: family interaction, parenting, emotional well‐being, physical/material well‐being, and disability‐related support. Response options were rated on a five‐point Likert scale: (1 = very dissatisfied, 2 = dissatisfied, 3 = neither satisfied nor dissatisfied, 4 = satisfied, and 5 = very satisfied). Similar to P‐C scales, a substantial portion of FQoL questions included resilience related constructs such as family interaction (Bonanno, 2004), parenting (Hayes & Watson, 2013; Kotera et al., 2021; Russell, Tomkunas, et al., 2022) and disability‐related support (Rutter, 1987, 1999).

We pooled COVID‐19 FQoL data with recently reported prepandemic data collected during 2018 (Bolbocean et al., 2021). We used identical methods to identify and recruit caregivers during prepandemic data collection and during the present COVID‐19 studies. Both studies relied on the same organizations in order to recruit families using patient registries. Thus, while the participants in both studies were recruited using identical methods and sample frames, these are not necessarily the same families. Moreover, unlike the COVID‐19 study, the prepandemic study did not collect sociodemographic information related to PMD, RTT, and *SYNGAP1*‐ID. However, the early study did provide sociodemographic information related to PMD, and *SYNGAP1*‐ID patients based on the organization's patients registries/databases (Bolbocean et al., 2021).

### FQoL prior and after the commencement of 2020 school year

We wanted to understand if systematic changes to COVID‐19 induced environments during autumn 2020—which brought the relaxation of previously imposed restrictions and the opening of schools—were associated with changes in the FQoL of caregivers. We aimed to compare FQoL outcomes during earlier and later phases of the outbreak across all diagnoses given that CDC guidance allowed of schools to reopen in September 2020 (CDC, 2020). There is evidence that the reopening of schools was beneficial for children's mental and physical health. We thus hypothesized that the caregiver's FQoL scores might experience an increase after the reopening of schools. In our dataset, the COVID‐19 FQoL data was collected between July 2020 and January 2021, and the response rate was not uniformly distributed across each day. However, the median participation rate was achieved on September 25, 2020. We defined the early COVID‐19 phase as the time between the start of data collection up to and including September 25, 2020, and the late COVID‐19 phase as the time after September 25, 2020 up to January 2021. This is consistent with findings from previous literature (Lordan et al., 2020; Sheikh et al., 2020). Finally, we used September 5th, 2020 to perform additional robustness checks.

### Association of COVID‐19 induced environments with FQoL and parent–child relationships

We assessed the association of COVID‐19 induced environments with FQoL and P‐C relationships measures. Both measures were transformed using linear transformation into scores ranging 0–100, 0 was the worst possible rating and 100 the best possible rating.

## EMPIRICAL ANALYSES

Differences in baseline characteristics, FQoL average scores before and during the COVID‐19 pandemic, differences in environments and PC interactions across diagnoses were ascertained using the ANOVA test for means, and Kruskal–Wallis test for medians. Fisher's exact test was used to assess differences for categorical variables. Differences between 2018 and during pandemic FQoL average scores were ascertained using the Student *t*‐test for unequal variances and nonparametric tests for medians (quantile regression test for the equality of medians; Conroy, 2012).

We employed a 1 to 1 random matching (Rothman, 2012; Stuart, 2010) to compare FQoL outcomes during earlier and later phase of the outbreak. We performed multivariable regressions analysis to explore the association between COVID‐19 induced environments, FQoL and P‐C average scores. We explored unadjusted and adjusted associations of COVID‐19 induced environments with FQoL and P‐C scores.^2^ To account for the presence of systematic differences and condition‐specific effects across diagnoses we corrected for the time‐invariant unobserved heterogeneity associated with each diagnosis by using an indicator for each of the disorders considered. Covariates included in the multivariable models were informed by previous theoretical and empirical models (Larsson et al., 2005; Olson et al., 2021; Thomas et al., 2012) and included: a child's age measured in years, a child's sex, and the caregiver's gender, race, education, marital status, as well as, the caregiver's annual income. We used the ordinary least squares (OLS) estimator to perform the multivariable regressions. Statistical analyses were conducted by using Stata 17.0 (Stata Corp, College Station, TX). The *p*‐values of 0.05 or less were considered statistically significant.

## RESULTS

### Baseline characteristics of parents

Table 1 reports baseline characteristics of the study's population across each diagnosis. Of the 230 parents, statistically significant differences across diagnosis (138 with PMD, 50 with *SYNGAP1*‐ID, and 42 with RTT) were found regarding the following variables: estimated total annual household income and the type of health insurance of the caregiver. In particular, the evidence shows that parents of *SYNGAP1*‐ID children were more affluent (over 61% had a total annual household income over $75,000 [USD]) followed by PMD with 49% and RTT with 42%. In addition, almost 50% had private health insurance followed by PMD with 35% and RTT with 31%. Parents of *SYNGAP1*‐ID children had the highest proportion of graduate or professional degrees with 49% followed by PMD 37% and RTT 32%. However, the overall evidence suggests that the majority of socioeconomic covariates were largely balanced across each of the three diagnoses.

**TABLE 1:** Demographic characteristics by child's diagnosis

	PMD	*SYNGAP1*‐ID	RTT	Total	*p*‐value
n (%)	138 (60.0)	50 (21.7)	42 (18.3)	230 (100.0)	
*Caregiver's gender*, n (%)					
Female	103 (77.4)	45 (90.0)	31 (83.8)	179 (81.4)	
Male	30 (22.6)	5 (10.0)	6 (16.2)	41 (18.6)	0.14
*Age*, n (%)					
35–44 years old	40 (30.1)	23 (46.0)	13 (35.1)	76 (34.5)	
45–54 years old	29 (21.8)	14 (28.0)	7 (18.9)	50 (22.7)	
55–64 years old	25 (18.8)	2 (4.0)	7 (18.9)	34 (15.5)	
65+ years old	2 (1.5)	0 (0.0)	2 (5.4)	4 (1.8)	
<35 years old	37 (27.8)	11 (22.0)	8 (21.6)	56 (25.5)	0.10
*Marital status*, n (%)					
Divorced	5 (5.0)	6 (12.8)	4 (10.8)	15 (8.2)	
Living with partner	7 (7.0)	3 (6.4)	1 (2.7)	11 (6.0)	
Married	83 (83.0)	37 (78.7)	31 (83.8)	151 (82.1)	
Never married	3 (3.0)	1 (2.1)	0 (0.0)	4 (2.2)	
Separated	2 (2.0)	0 (0.0)	1 (2.7)	3 (1.6)	0.65
*Race/ethnicity*, n (%)					
American Indian or Alaska Native	1 (1.0)	0 (0.0)	0 (0.0)	1 (0.5)	
Asian	4 (4.0)	2 (4.3)	3 (8.3)	9 (4.9)	
Black or African American	1 (1.0)	2 (4.3)	0 (0.0)	3 (1.6)	
Other	6 (5.9)	2 (4.3)	1 (2.8)	9 (4.9)	
White	89 (88.1)	41 (87.2)	32 (88.9)	162 (88.0)	0.72
*Estimated total annual household income*, n (%)					
Less than or equal to $34,999	27 (27.6)	8 (17.0)	5 (13.9)	40 (22.1)	
$35,000–$74,999	23 (23.5)	10 (21.3)	16 (44.4)	49 (27.1)	
≥ $75,000	48 (49.0)	29 (61.7)	15 (41.7)	92 (50.8)	0.04
*Health insurance status*, n (%)					
Medicaid and private	34 (34.3)	16 (34.0)	13 (36.1)	63 (34.6)	
Medicaid and/or Medicare only	15 (15.2)	7 (14.9)	12 (33.3)	34 (18.7)	
No health insurance	15 (15.2)	1 (2.1)	0 (0.0)	16 (8.8)	
Private only (military, employer, private, other)	35 (35.4)	23 (48.9)	11 (30.6)	69 (37.9)	0.01
*Primary language spoken in home*, n (%)					
English	78 (77.2)	38 (80.9)	34 (91.9)	150 (81.1)	
Other	18 (17.8)	7 (14.9)	2 (5.4)	27 (14.6)	
Spanish	5 (5.0)	2 (4.3)	1 (2.7)	8 (4.3)	0.42
*Level of education*, n (%)					
< High school	11 (11.0)	3 (6.4)	3 (8.1)	17 (9.2)	
College degree	38 (38.0)	15 (31.9)	13 (35.1)	66 (35.9)	
Graduate or professional degree	37 (37.0)	23 (48.9)	12 (32.4)	72 (39.1)	
High school or GED equivalent	8 (8.0)	0 (0.0)	7 (18.9)	15 (8.2)	
Technical school	6 (6.0)	6 (12.8)	2 (5.4)	14 (7.6)	0.08
*Relationship to the child*, n (%)					
Father	21 (15.8)	4 (8.0)	6 (16.2)	31 (14.1)	
Grandfather	0 (0.0)	0 (0.0)	1 (2.7)	1 (0.5)	
Grandmother	2 (1.5)	0 (0.0)	0 (0.0)	2 (0.9)	
Mother	108 (81.2)	45 (90.0)	30 (81.1)	183 (83.2)	
Other	2 (1.5)	1 (2.0)	0 (0.0)	3 (1.4)	0.34
*Smoking status*, n (%)					
No	97 (97.0)	43 (91.5)	35 (97.2)	175 (95.6)	
Yes	3 (3.0)	4 (8.5)	1 (2.8)	8 (4.4)	0.27
*COVID‐19 status*, n (%)					
No	95 (97.9)	41 (95.3)	28 (96.6)	164 (97.0)	
Yes	2 (2.1)	2 (4.7)	1 (3.4)	5 (3.0)	0.70
*Health status during the COVID‐19 outbreak*, n (%)					
Average	13 (13.5)	9 (20.9)	9 (31.0)	31 (18.5)	
Excellent	35 (36.5)	13 (30.2)	4 (13.8)	52 (31.0)	
Good	42 (43.8)	19 (44.2)	13 (44.8)	74 (44.0)	
Poor	6 (6.3)	2 (4.7)	3 (10.3)	11 (6.5)	0.20
*Participation in WIC program*, n (%)					
No	79 (79.8)	40 (85.1)	24 (66.7)	143 (78.6)	
Yes	20 (20.2)	7 (14.9)	12 (33.3)	39 (21.4)	0.12
*Child's age in years*, mean (SD)	11.05 (5.71)	11.78 (5.02)	12.30 (4.57)	11.48 (5.33)	0.49
*Child's age at diagnosis in years*, mean (SD)	9.44 (4.47)	8.42 (4.76)	9.56 (4.54)	9.15 (4.55)	0.55
*Child's sex*, n (%)					
Female	46 (46.9)	24 (51.1)	29 (96.7)	99 (56.6)	
Male	52 (53.1)	23 (48.9)	1 (3.3)	76 (43.4)	0.00

*Note*: For child's age and age at diagnosis *p*‐value is based on an ANOVA test. For other variables *p*‐value is based on Fisher's exact test. WIC stands for The Special Supplemental Nutrition Program for Women, Infants, and Children. Additional details regarding children's characteristics can be found in (Bolbocean et al., 2022).

### Parental environments during COVID‐19

Table 2 shows the distribution of covariates which describe the environments parents faced during COVID‐19 across each diagnosis. Results show significant differences across PMD, *SYNGAP1‐*ID, and RTT when parents were asked if they were able to maintain a predictable routine during COVID‐19. Overall, 15% of caregivers reported an inability to maintain a predictable routine during the COVID‐19 pandemic. This proportion was the highest among parents of PMD children (23%), followed by RTT parents (7%), and *SYNGAP1*‐ID (5%).

**TABLE 2:** Parental environments during COVID‐19 by child's diagnosis

	PMD	*SYNGAP1*‐ID	RTT	Total	*p*‐value
n (%)	138 (60.0)	50 (21.7)	42 (18.3)	230 (100.0)	
*Have you had childcare help during the “shelter in place” orders?*, n (%)					
No	54 (56.3)	17 (39.5)	16 (55.2)	87 (51.8)	
Prefer not to answer	1 (1.0)	0 (0.0)	1 (3.4)	2 (1.2)	
Yes	41 (42.7)	26 (60.5)	12 (41.4)	79 (47.0)	0.22
*Who has helped you during the “shelter in place” orders with childcare?*, n (%)					
Family member other than partner	11(30.5)	10 (28.6)	3(24.9)	24 (28.9)	
Friend	6 (16.7)	4 (11.5)	3 (24.9)	13 (15.6)	
Other/Out of home care	19 (52.9)	21 (60.0)	6 (49.8)	46 (55.4)	0.09
*How much the COVID‐19 outbreak has changed your access to medical care?*, n (%)					
Mild.	24 (29.6)	19 (44.2)	9 (32.1)	52 (34.2)	
Moderate. Delays or cancellations in appointments.	28 (34.6)	13 (30.2)	10 (35.7)	51 (33.6)	
No change.	27 (33.3)	6 (14.0)	8 (28.6)	41 (27.0)	
Severe. Unable to access care resulting in moderate/severe impact on health.	2 (2.5)	5 (11.6)	1 (3.6)	8 (5.3)	0.10
*How much the COVID‐19 has changed your access to mental health treatments?*, n (%)					
Mild.	19 (25.7)	15 (38.5)	8 (30.8)	42 (30.2)	
Moderate. Delays or cancellations in appointments.	12 (16.2)	7 (17.9)	2 (7.7)	21 (15.1)	
No change.	36 (48.6)	13 (33.3)	13 (50.0)	62 (44.6)	
Severe. Unable to access care resulting in moderate/severe impact on health.	7 (9.5)	4 (10.3)	3 (11.5)	14 (10.1)	0.64
*During COVID‐19 the number of people living in home changed?*, n (%)					
No, there are the same number of people in my home	66 (81.5)	37 (86.0)	23 (82.1)	126 (82.9)	
Yes, there are fewer people in my home	4 (4.9)	2 (4.7)	1 (3.6)	7 (4.6)	
Yes, there are more people in my home	11 (13.6)	4 (9.3)	4 (14.3)	19 (12.5)	0.96
*Were you able to maintain a predictable routine during COVID‐19?*, n (%)					
Most of the time	38 (48.1)	17 (42.5)	14 (50.0)	69 (46.9)	
Not at all	18 (22.8)	2 (5.0)	2 (7.1)	22 (15.0)	
Prefer not to answer	1 (1.3)	0 (0.0)	0 (0.0)	1 (0.7)	
Somewhat	22 (27.8)	21 (52.5)	12 (42.9)	55 (37.4)	0.05
*How much stress have you experienced due to potential exposure to COVID‐19?*, n (%)					
Do not know	0 (0.0)	1 (2.3)	1 (3.6)	2 (1.3)	
Not at all stressed	22 (27.2)	3 (7.0)	3 (10.7)	28 (18.4)	
Somewhat stressed	32 (39.5)	23 (53.5)	10 (35.7)	65 (42.8)	
Very stressed	27 (33.3)	16 (37.2)	14 (50.0)	57 (37.5)	0.04
*How difficult has it been to pay for basic expenses?*, n (%)					
Not at all difficult	54 (67.5)	23 (53.5)	14 (50.0)	91 (60.3)	
Prefer not to answer	0 (0.0)	3 (7.0)	0 (0.0)	3 (2.0)	
Somewhat difficult	17 (21.3)	14 (32.6)	9 (32.1)	40 (26.5)	
Very difficult	9 (11.3)	3 (7.0)	5 (17.9)	17 (11.3)	0.05
*How family finances worked out at the end of the month?*, n (%)					
Just enough to make ends meet	24 (30.0)	18 (41.9)	13 (46.4)	55 (36.4)	
Not enough to make ends meet	7 (8.8)	2 (4.7)	4 (14.3)	13 (8.6)	
Prefer not to answer	2 (2.5)	3 (7.0)	0 (0.0)	5 (3.3)	
Some money left over	47 (58.8)	20 (46.5)	11 (39.3)	78 (51.7)	0.20

*Note*: *p*‐value is based on Fisher's exact.

Results also show significant differences across diagnoses when we measured experiences of family stress concerning potential exposure to COVID‐19 at work or while commuting to and from work, as well as, when measuring a caretaker's ability to provide basic needs such as food, housing, and medical care. Thus, more than 80% of parents reported being somewhat stressed or very stressed due to potential exposure to COVID‐19 with the greatest proportion of highly stressed parents recorded within RTT. Over half of caregivers reported that some money was left over at the end of the month during the pandemic, however, the overall proportion of parents who did not have enough to make ends meet was close to 9%. This was the highest within RTT (15%). Notably, 47% of caregivers reported having help with childcare during “shelter in place” orders, and over 55% of parents reported that their help came from someone outside of their home.

Reported evidence in Table 2 shows that overall, 73% of parents reported that COVID‐19 had changed their healthcare access: mild change was reported by 34%, moderate change, that is, delays or cancellations in appointments reported by 34%, and severe changes defined as the inability to access care which resulted in moderate/severe impact on health, was reported by 5%. Over half of caregivers reported that COVID‐19 had changed their access to mental health treatments: severe change was reported by 10% of parents, moderate change reported by 15% and mild change reported by 30%.

### FQoL scores prior and during the first year of the COVID‐19 pandemic

Table 3 reports average FQoL scores for time periods before and during the pandemic cohorts across domains using the combined sample and disaggregated by the diagnosis. The evidence within the combined sample has shown that there were no statistically significant differences in average FQoL scores between times periods before and during COVID‐19. Interestingly, the data shows that families of *SYNGAP1*‐ID patients had a minor increase related to family interaction score and emotional score. However, no differences were detected within families of PMD and RTT patients between data gathered before and during COVID‐19. Additional details can be found in Appendix B. For robustness checks we utilized a 1 to 1 random matching (Rothman, 2012; Stuart, 2010) to compare the average FQoL scores collected during 2018 and during the first year of the pandemic. The evidence of this analysis showed that there were no statistically significant differences in FQoL between the time periods before and during COVID‐19.

**TABLE 3:** FQoL outcomes pre and during COVID‐19 pandemic

	Before‐COVID19	During COVID‐19	Total	*p*‐value
*All diagnoses*	391 (56.6)	230 (43.4)	621 (100.0)	
Family interaction score, mean (95% CI)	3.30 (3.20; 3.39)	3.37 (3.21; 3.52)	3.31 (3.23; 3.39)	0.46
Family interaction score, median (IQI)	3.17 (2.67; 4.00)	3.33 (2.67; 4.00)	3.17 (2.67; 4.00)	0.67
Parenting score, mean (95% CI)	3.04 (2.96; 3.13)	3.06 (2.92; 3.20)	3.05 (2.98; 3.12)	0.83
Parenting score, median (IQI)	3.00 (2.50; 3.50)	2.83 (2.50; 3.50)	3.00 (2.50; 3.50)	0.75
Emotional score, mean (95% CI)	2.64 (2.56; 2.73)	2.76 (2.61; 2.92)	2.68 (2.60; 2.75)	0.19
Emotional Score, median (IQI)	2.50 (2.00; 3.25)	2.75 (2.25; 3.25)	2.50 (2.00; 3.25)	0.27
Physical well‐being score, mean (95% CI)	3.67 (3.58; 3.76)	3.64 (3.49; 3.79)	3.66 (3.58; 3.74)	0.79
Physical well‐being score, median (IQI)	3.60 (3.00; 4.60)	3.40 (3.00; 4.60)	3.60 (3.00; 4.60)	0.83
Disability support score, mean (95% CI)	3.14 (3.04; 3.24)	3.14 (2.97; 3.31)	3.14 (3.06; 3.23)	0.97
Disability support score, median (IQI)	3.00 (2.50; 3.75)	3.00 (2.50; 3.75)	3.00 (2.50; 3.75)	0.90
*PMD*	213 (60.7)	138 (39.3)	351 (100.0)	
Family interaction score, mean (95% CI)	3.33 (3.21; 3.45)	3.36 (3.17; 3.55)	3.34 (3.24; 3.44)	0.82
Family interaction score, median (IQI)	3.17 (2.67; 4.00)	3.33 (2.67; 3.83)	3.17 (2.67; 4.00)	0.83
Parenting score, mean (95% CI)	3.06 (2.95; 3.18)	3.05 (2.86; 3.25)	3.06 (2.96; 3.16)	0.95
Parenting score, median (IQI)	3.00 (2.50; 3.50)	2.83 (2.50; 3.50)	3.00 (2.50; 3.50)	0.70
Emotional score, mean (95% CI)	2.67 (2.55; 2.80)	2.80 (2.60; 3.01)	2.70 (2.60; 2.81)	0.32
Emotional score, median (IQI)	2.50 (2.00; 3.25)	2.75 (2.25; 3.25)	2.75 (2.00; 3.25)	0.31
Physical well‐being score, mean (95% CI)	3.74 (3.62; 3.86)	3.64 (3.43; 3.85)	3.72 (3.61; 3.82)	0.41
Physical well‐being score, median (IQI)	3.60 (3.00; 4.60)	3.40 (3.00; 4.60)	3.60 (3.00; 4.60)	0.41
Disability support score, mean (95% CI)	3.18 (3.05; 3.31)	3.26 (3.01; 3.52)	3.20 (3.08; 3.31)	0.55
Disability support score, median (IQI)	3.00 (2.50; 4.00)	3.00 (2.50; 4.00)	3.00 (2.50; 4.00)	0.61
*SYNGAP1*‐ID	30 (37.5)	50 (62.5)	80 (100.0)	
Family interaction score, mean (95% CI)	2.69 (2.36; 3.03)	3.29 (2.98; 3.60)	3.04 (2.80; 3.27)	0.01
Family interaction score, median (IQI)	2.67 (2.25; 3.42)	3.00 (2.67; 3.92)	2.83 (2.33; 3.67)	0.03
Parenting score, mean (95% CI)	2.58 (2.30; 2.85)	2.96 (2.70; 3.22)	2.80 (2.60; 2.99)	0.06
Parenting score, median (IQI)	2.50 (2.17; 3.00)	2.83 (2.33; 3.25)	2.67 (2.33; 3.00)	0.08
Emotional score, mean (95% CI)	2.27 (2.03; 2.50)	2.67 (2.37; 2.97)	2.50 (2.30; 2.71)	0.05
Emotional score, median (IQI)	2.25 (2.00; 2.75)	2.50 (2.13; 3.00)	2.50 (2.00; 2.75)	0.08
Physical well‐being score, mean (95% CI)	3.49 (3.16; 3.83)	3.56 (3.30; 3.82)	3.53 (3.33; 3.74)	0.76
Physical well‐being score, median (IQI)	3.40 (2.75; 4.20)	3.40 (3.00; 4.20)	3.40 (2.80; 4.20)	0.63
Disability support score, mean (95% CI)	2.80 (2.44; 3.17)	2.93 (2.62; 3.23)	2.87 (2.64; 3.11)	0.61
Disability support score, median (IQI)	2.75 (2.00; 3.50)	2.50 (2.25; 3.50)	2.75 (2.25; 3.50)	0.71
*RTT*	148 (77.9)	42 (22.1)	190 (100.0)	
Family interaction score, mean (95% CI)	3.37 (3.21; 3.53)	3.53 (3.06; 4.00)	3.39 (3.24; 3.54)	0.47
Family interaction score, median (IQI)	3.17 (2.67; 4.33)	3.67 (2.33; 5.00)	3.17 (2.67; 4.33)	0.57
Parenting score, mean (95% CI)	3.11 (2.98; 3.25)	3.34 (2.96; 3.72)	3.14 (3.01; 3.27)	0.23
Parenting score, median (IQI)	3.00 (2.50; 3.67)	3.00 (2.67; 4.17)	3.00 (2.50; 3.67)	0.37
Emotional score, mean (95% CI)	2.68 (2.53; 2.82)	2.97 (2.54; 3.39)	2.72 (2.58; 2.86)	0.16
Emotional score, median (IQI)	2.63 (2.00; 3.25)	3.00 (2.50; 4.00)	2.75 (2.00; 3.25)	0.20
Physical well‐being score, mean (95% CI)	3.60 (3.44; 3.75)	3.84 (3.43; 4.25)	3.63 (3.49; 3.78)	0.26
Physical well‐being score, median (IQI)	3.50 (2.80; 4.60)	4.20 (3.00; 4.60)	3.60 (2.80; 4.60)	0.24
Disability support score, mean (95% CI)	3.15 (2.99; 3.31)	3.25 (2.82; 3.68)	3.17 (3.02; 3.31)	0.65
Disability support score, median (IQI)	3.00 (2.50; 3.75)	3.00 (2.50; 4.00)	3.00 (2.50; 3.75)	0.71

*Note*: When mean is reported the *p*‐value is based on a *t*‐test of unequal variances; when median is reported the *p*‐value is based on a Kruskal Wallis test.

Abbreviations: CI, confidence interval; IQI, interquartile interval.

### FQoL prior and after the commencement of 2020 school year: Evidence from PMD caregivers

To ascertain the impact of early versus late COVID‐19 on average FQoL scores, and to control for heterogeneity across clinical profiles, we randomly matched observations before and after September 25th according to clinical diagnosis. From the start of data collection before and on September 25th (55.7%) respondents completed the survey. Given the sample sizes across diagnoses shown in Table 1, we were only able to match PMD parents.^3^ Table 4 reports results from comparing FQoL outcomes by date of responses among caregivers of PMD children. Results show that FQoL scores for families who completed the measure prior to September 25th did not differ from scores of those who completed surveys after this date.

**TABLE 4:** FQoL outcomes early versus late COVID‐19: Matched analysis for PMD patients

	Before September 25	After September 25	Difference	SD	Lower 95% CI	Upper 95% CI	*p*‐value
N (%)	65 (50.0)	65 (50.0)					
Family interaction score, mean (95% CI)	3.32 (3.06; 3.58)	3.39 (3.11; 3.68)	0.08	0.20	−0.32	0.48	0.70
Parenting score, mean (95% CI)	3.00 (2.70; 3.30)	3.11 (2.83; 3.39)	0.11	0.21	−0.31	0.53	0.59
Emotional score, mean (95% CI)	2.82 (2.49; 3.15)	2.79 (2.51; 3.06)	−0.03	0.22	−0.47	0.40	0.87
Physical well‐being score, mean (95% CI)	3.52 (3.21; 3.84)	3.74 (3.45; 4.04)	0.22	0.22	−0.22	0.66	0.32
Disability support score, mean (95% CI)	3.29 (2.96; 3.63)	3.22 (2.83; 3.61)	−0.07	0.27	−0.62	0.47	0.78

*Note*: *p*‐value is based on Student *t*‐test for unequal variances.

### Parent–child interactions during COVID‐19

Table 5 shows the distribution of covariates which describe PC interactions during COVID‐19 across PMD, *SYNGAP1*‐ID, and RTT. We found statistically significant differences across diagnoses when we asked if children were turning to their caretakers with personal problems, as well as, how often a child depended on the caretaker's help, advice, or sympathy. Results were then paired with the caretaker's role in cheering up the child during the pandemic. Furthermore, statistically significant differences were found when we asked caretakers if they were quarreling or disagreeing with their child as a result of the COVID‐19 pandemic.

**TABLE 5:** Parent–child relationship during COVID‐19 by child's diagnosis

	PMD	*SYNGAP1*‐ID	RTT	Total	*p*‐value
n (%)	138 (60.0)	50 (21.7)	42 (18.3)	230 (100.0)	
*How often do you spend fun time with your child?*, n (%)					
Always/Extremely	7 (8.6)	6 (14.0)	6 (21.4)	19 (12.5)	
Often/Very much	39 (48.1)	20 (46.5)	12 (42.9)	71 (46.7)	
Sometimes	26 (32.1)	13 (30.2)	8 (28.6)	47 (30.9)	
Seldom/Not too much	8 (9.9)	4 (9.3)	0 (0.0)	12 (7.9)	
Never/Hardly at all	1 (1.2)	0 (0.0)	2 (7.1)	3 (2.0)	0.22
*How happy are you with your relationship with your child?*, n (%)					
Always/Extremely	23 (28.4)	9 (20.9)	14 (50.0)	46 (30.3)	
Often/Very much	31 (38.3)	20 (46.5)	8 (28.6)	59 (38.8)	
Seldom/Not too much	5 (6.2)	1 (2.3)	1 (3.6)	7 (4.6)	
Sometimes	21 (25.9)	13 (30.2)	5 (17.9)	39 (25.7)	0.32
Never/Hardly at all	1 (1.2)	0 (0.0)	0 (0.0)	1 (0.7)	
*How often do you and your child disagree and quarrel with one another?*, n (%)					
Always/Extremely	0 (0.0)	0 (0.0)	1 (3.6)	1 (0.7)	
Often/Very much	7 (8.8)	5 (11.6)	2 (7.1)	14 (9.3)	
Seldom/Not too much	13 (16.3)	11 (25.6)	3 (10.7)	27 (17.9)	
Sometimes	17 (21.3)	18 (41.9)	7 (25.0)	42 (27.8)	
Never/Hardly at all 43 (53.8)	9 (20.9)	15 (53.6)	67 (44.4)	0.01	
*How often does your child turn to you for support with personal problems?*, n (%)					
Always/Extremely	15 (18.8)	15 (34.9)	8 (29.6)	38 (25.3)	
Often/Very much	18 (22.5)	20 (46.5)	10 (37.0)	48 (32.0)	
Seldom/Not too much	7 (8.8)	3 (7.0)	1 (3.7)	11 (7.3)	
Sometimes	13 (16.3)	4 (9.3)	4 (14.8)	21 (14.0)	
Never/Hardly at all	27 (33.8)	1 (2.3)	4 (14.8)	32 (21.3)	0.00
*How often do you point out his/her faults or put your child down?*, n (%)					
Often/Very much	1 (1.3)	1 (2.3)	0 (0.0)	2 (1.3)	
Seldom/Not too much	16 (20.3)	16 (37.2)	2 (7.1)	34 (22.7)	
Sometimes	7 (8.9)	6 (14.0)	3 (10.7)	16 (10.7)	
Never/Hardly at all	55 (69.6)	20 (46.5)	23 (82.1)	98 (65.3)	0.06
*How often do you and your child go places and do things together?*, n (%)					
Always/Extremely	8 (9.9)	5 (11.6)	4 (14.3)	17 (11.2)	
Often/Very much	33 (40.7)	16 (37.2)	10 (35.7)	59 (38.8)	
Seldom/Not too much	9 (11.1)	6 (14.0)	4 (14.3)	19 (12.5)	
Sometimes	22 (27.2)	9 (20.9)	7 (25.0)	38 (25.0)	
Never/Hardly at all	9 (11.1)	7 (16.3)	3 (10.7)	19 (12.5)	0.98
*How often do you and your child get mad at or get into fights with one another?*, n (%)					
Often/Very much	2 (2.5)	1 (2.3)	1 (3.7)	4 (2.7)	
Seldom/Not too much	14 (17.5)	17 (39.5)	6 (22.2)	37 (24.7)	
Sometimes	10 (12.5)	8 (18.6)	3 (11.1)	21 (14.0)	
Never/Hardly at all	54 (67.5)	17 (39.5)	17 (63.0)	88 (58.7)	0.11
*How often does your child depend on your for help, advice, or sympathy?*, n (%)					
Always/Extremely	22 (27.2)	21 (48.8)	19 (70.4)	62 (41.1)	
Often/Very much	34 (42.0)	13 (30.2)	6 (22.2)	53 (35.1)	
Seldom/Not too much	2 (2.5)	2 (4.7)	0 (0.0)	4 (2.6)	
Sometimes	12 (14.8)	6 (14.0)	1 (3.7)	19 (12.6)	
Never/Hardly at all	11 (13.6)	1 (2.3)	1 (3.7)	13 (8.6)	0.01
*How often do you criticize your child?*, n (%)					
Never/Hardly at all	59 (72.8)	28 (66.7)	24 (88.9)	111 (74.0)	
Seldom/Not too much	16 (19.8)	9 (21.4)	2 (7.4)	27 (18.0)	
Sometimes	6 (7.4)	5 (11.9)	1 (3.7)	12 (8.0)	0.32
*How often do you play around and have fun with your child?*, n (%)					
Always/Extremely	9 (11.3)	5 (11.9)	4 (14.3)	18 (12.0)	
Often/Very much	38 (47.5)	21 (50.0)	11 (39.3)	70 (46.7)	
Seldom/Not too much	10 (12.5)	3 (7.1)	1 (3.6)	14 (9.3)	
Sometimes	19 (23.8)	13 (31.0)	12 (42.9)	44 (29.3)	
Never/Hardly at all	4 (5.0)	0 (0.0)	0 (0.0)	4 (2.7)	0.36
*How often do you and your child argue with one another?*, n (%)					
Always/Extremely	1 (1.2)	0 (0.0)	0 (0.0)	1 (0.7)	
Often/Very much	2 (2.5)	3 (7.1)	1 (3.7)	6 (4.0)	
Seldom/Not too much	12 (14.8)	12 (28.6)	4 (14.8)	28 (18.7)	
Sometimes	3 (3.7)	7 (16.7)	2 (7.4)	12 (8.0)	
Never/Hardly at all	63 (77.8)	20 (47.6)	20 (74.1)	103 (68.7)	0.06
*When you child is feeling down how often does he/she depend on you to cheer things up?*, n (%)					
Always/Extremely	14 (17.5)	17 (39.5)	14 (51.9)	45 (30.0)	
Often/Very much	26 (32.5)	18 (41.9)	6 (22.2)	50 (33.3)	
Seldom/Not too much	8 (10.0)	1 (2.3)	1 (3.7)	10 (6.7)	
Sometimes	16 (20.0)	6 (14.0)	5 (18.5)	27 (18.0)	
Never/Hardly at all	16 (20.0)	1 (2.3)	1 (3.7)	18 (12.0)	0.00
*How often do you say mean or harsh things to your child?*, n (%)					
Never/Hardly at all	63 (78.8)	27 (62.8)	24 (85.7)	114 (75.5)	
Seldom/Not too much	11 (13.8)	12 (27.9)	3 (10.7)	26 (17.2)	
Sometimes	6 (7.5)	4 (9.3)	1 (3.6)	11 (7.3)	0.18
*How connected have you felt to your child during the COVID‐19 outbreak?*, n (%)					
A lot more connected than before the outbreak	20 (25.0)	12 (28.6)	9 (32.1)	41 (27.3)	
A little more connected than before the outbreak	13 (16.3)	11 (26.2)	2 (7.1)	26 (17.3)	
A little less connected than before the outbreak	5 (6.3)	3 (7.1)	4 (14.3)	12 (8.0)	
A lot less connected than before the outbreak	1 (1.3)	0 (0.0)	1 (3.6)	2 (1.3)	
Just as connected than before the outbreak	41 (51.2)	16 (38.1)	12 (42.9)	69 (46.0)	0.39

*Note*: *p*‐value is based on Fisher's exact test.

When considering the presence of conflict in a child with syndromic autism, our results indicated that the root of that conflict is often not the PC relationship with 44.4% of respondents reporting that they never/hardly at all disagree or quarrel with one another. Parents of PMD children reported the lowest levels of disagreement or quarreling with 53.8% reporting never/hardly at all as compared with 53.6% with RTT and 20.9% from *SYNGAP1*‐ID. These trends continued with 68.7% of parents and caretakers responding that they never/hardly at all argue with their children, 75.5% never/hardly at all say mean or harsh things to their children, 74% answered never/hardly at all criticize their children. Finally, 65.3% of respondents answered that they never/hardly at all put their child down.

When asked how happy parents were with their relationship with their child an overwhelming majority reported being happy always (30.3%) or often/very often (38.8%) in their interactions. Across each diagnosis we consistency saw in this area a combination of being happy always or often/very often in the relationship with 66.7% with PMD PC relationships, 67.4% among *SYNGAP1*‐ID PC relationships, and 78.6% among RTT PC relationships. Results show that support seeking was also high among *SYNGAP1*‐ID children with 34.9% reporting that they always seek support and 46.5% seeking support often. These results also remained high among RTT children with 29.6% always and 37% often seeking support. On the other hand, data showed that PMD children had lower support seeking behaviors with 33.8% never/hardly and 16.3% sometimes seeking support. Across all diagnosis 59.2% of respondents reported spending fun time with their child always or often, and 50% responding that they always or often go places and do things together with their children. The evidence indicated that 41.1% of children still depended on their parents for help, advice, or sympathy during the pandemic, and 30% of children always relied on their parent paired with 33.3% who relied on them often during the pandemic to cheer them up when they were feeling down. The parents of children diagnosed with RTT reported feeling a lot more connected during the outbreak than before (32.1%) followed by *SYNGAP1*‐ID with 28.6% and PMD with 25%.

### The association between COVID‐19 factors with FQoL and P‐C average

### Scores

Table 6 reports results from OLS regression models which assessed the unadjusted and adjusted associations between COVID‐19 induced environments with average FQoL and P‐C average scores.^4^ The evidence suggests that COVID‐19 variables were statistically significant predictors of average FQoL scores. In particular, the indicator variable which denoted that a caregiver recorded a severe or moderate change in access to healthcare services during the pandemic was associated with a decrease of −12.76 (95% CI: −20.23, −5.29) points in average FQoL scores. A caregiver's ability to maintain a predictable routine or schedule during COVID‐19 was associated with 10.72 (95% CI: 6.55, 14.90) points in average FQoL scores. Finally, the variable which indicated that a child was somewhat successful or very successful in completing home school assignments was associated with an increase of 11.15 (95% CI: −4.94, 27.24) in average FQoL scores.

**TABLE 6:** Multivariable analyses of FQoL scores and parent–child relationship scores during COVID‐19 (OLS regression)

		Unadjusted					Adjusted				
Outcome	COVID‐19 factors	*β*	SE	Lower 95% CI	Upper 95% CI	p‐value	*β*	SE	Lower 95% CI	Upper 95% CI	p‐value
FQoL scores	Finance	5.44	3.29	−8.71	19.58	0.10	5.88	5.00	−15.62	27.37	0.24
	Health access	−12.72	1.84	−20.63	−4.80	0.00	−12.76	1.74	−20.23	−5.29	0.00
	Routine	9.36	2.32	−0.60	19.32	0.00	10.72	0.97	6.55	14.90	0.00
	Home school	10.27	2.72	−1.44	21.98	0.00	11.15	3.74	−4.94	27.24	0.00
P‐C scores	Finance	−0.95	0.67	−3.82	1.93	0.16	0.43	0.67	−2.47	3.32	0.53
	Health access	−0.08	1.61	−7.01	6.85	0.96	−0.30	1.18	−5.38	4.78	0.80
	Routine	3.39	1.21	−1.80	8.57	0.00	4.31	1.58	−2.47	11.09	0.01
	Home school	5.54	0.24	4.51	6.57	0.00	6.20	1.47	−0.12	12.52	0.00
	Controls			No					Yes		

*Note*: Variable “Finance” is an indicator which denotes that some money was left over at the end of the month during COVID‐19 pandemic. Variable “Health Access” is an indicator variable which denotes that caregiver recorded a severe (“unable to access needed care required”) or moderate (“delays or cancellations in appointments”) change to healthcare services during COVID‐19 outbreak. Variable “Routine” indicates that caregiver was able to maintain a predictable routine or schedule during COVID‐19 somewhat or most of the time. Variable “Home School” indicates that caregiver that during COVID‐19 the child was somewhat successful or very successful in completing the recommended home school assignments. Controls stand for control variables included: child's age measured in years, child's sex, caregiver's gender, indicator for race (white), indicator for graduate of post‐graduate education, indicator for married status, indicator for higher education, indicator for income ≥ $50.000 US.

Abbreviations: CI, confidence interval; FQoL, family quality of life; P‐C, parent–child relationship; SE, standard error.

Results from our analyses that examined the association between COVID‐19 parental environments and average P‐C scores were similar in magnitude and direction to average FQoL scores reported in the previous paragraph. In particular, a caregiver's ability to maintain a predictable routine or schedule during COVID‐19 was associated with 4.31 (95% CI: −2.47, 11.09) points in average P‐C scores. Furthermore, an indicator for a child's somewhat successful or very successful completion of home school assignments was associated with 6.20 (95% CI: −0.12, 12.52) in P‐C average scores. However, in equations designed to predict average FQoL and average P‐C scores the indicator which denoted that some money was left over at the end of the month during the pandemic was not significant. Interestingly, changes to a caregivers' access to healthcare was not found to be associated with average P‐C scores.

## DISCUSSION

In this study, we documented parental environments during the first year of the COVID‐19 pandemic of autistic and ID children diagnosed with PMD, *SYNGAP1*‐ID, and RTT. The availability of FQoL data primarily collected during 2018 and the first year of the pandemic allowed us to estimate the impact of COVID‐19 on a family's quality of life outcomes and compare FQoL outcomes during earlier and later phases of the outbreak. Furthermore, we ascertained the PC relationships and overall parenthood experiences during COVID‐19, and estimated the association between pandemic induced factors and parental experiences among syndromic autistic families.

Consistent with previous research we found that the COVID‐19 pandemic significantly changed families' routines (Cluver et al., 2020; Prime et al., 2020). Thus, results showed that the COVID19 pandemic had significantly changed caregivers' environments as 73% of parents reported that COVID‐19 had changed their healthcare access. Over half of caregivers reported that the pandemic had changed their mental health treatments or access to medical care.

The evidence in this study consistently demonstrated that within the combined sample there were no statistically significant differences in average FQoL scores between 2018 and during the first year of COVID‐19. Furthermore, no differences were detected in FQoL resilience related constructs such as family interaction (Bonanno, 2004), or parenting (Hayes & Watson, 2013; Kotera et al., 2021; Russell, Tomkunas, et al., 2022) and disability‐related support (Rutter, 1987, 1999) for PMD and RTT families. Interestingly, an increase in the average FQoL resilience related constructs were recorded for *SYNGAP1*‐ID families. Furthermore, no differences in average FQoL scores for parents of autistic children were detected between earlier and later phase of the first year of the pandemic.

Despite significant pandemic‐induced challenges for parents, our results indicated a strong bond between parents and their children during this time as an overwhelming majority of parents reported being always or often/very often happy in their interactions with their children. The overall high ratings of relational satisfaction are supported in other questions within our dataset that focus on levels of social support experienced between parents and their children during the pandemic across each diagnosis. The presence of a supportive environment and positive parenting is shown by the high rates of support seeking behaviors from children to their parents during the first year of the pandemic. The supportive environment of the PC relationship during COVID‐19 is also shown in responses centered on having fun during their time together as almost 2/3 of respondents reported spending fun time with their child always or often, and 1/2 of respondents stating that they always or often go places and do things together with their children. Furthermore, results have shown that emotional support remained a top support seeking behavior from autistic children during the pandemic.

The low presence of conflict reported in our data, coupled with the high‐levels of support seeking and quality interactions between parents and their children during the pandemic are present within our findings and overall indicate resilience and positive parenting. The data also showed that parents across diagnosis reported some increase in the feelings of connection with their children during the outbreak. Overall, the PC relationships showed incredible strength and our parent participants showed enormous resilience in caretaking during a stressful period.

Results also demonstrated the presence of resilience in the context of stressful COVID‐19 environments across PMD, SYNGAP1‐ID and RTT families. First, the evidence consistently demonstrated that COVID‐19 had adversely impacted parental environments. Surprisingly, overall only 15% of caregivers reported an inability to maintain a predictable routine during the COVID‐19 pandemic which was well below the rates reported in the literature during similar periods (Adams et al., 2021; Cluver et al., 2020; Liu et al., 2021; Russell et al., 2020; Russell, Hutchison, et al., 2022). The differences in these rates suggest that parents in our sample displayed incredible resilience and stoicism. Second, the overall evidence in this study shows that there were no statistically significant differences in FQoL resilience related constructs across repeated surveys primarily collected during 2018 and during the first year of COVID‐19. Furthermore, we found that average FQoL resilience related scores did not change between early versus late stages of the first year of the pandemic. During stressful times, such as the COVID‐19 pandemic, one might expect to see a reported increase in conflict communication within households, however, surprisingly our results demonstrated that conflict in communicatively expressed interactions remained low. In addition, results indicated that the root of that conflict was often not the PC relationship, and that close to half of respondents reported that they never/hardly at all disagreed or quarreled with one another. At the same time, the COVID‐19 pandemic was positively associated with PC bonding and relational satisfaction. Specifically, across each diagnosis we found that parents reported some increase in the feelings of connection with their children during the outbreak and emotional support remained a top support seeking behavior from children.

Overall evidence in this study suggests that resilience is the likely mechanism that explains positive PC outcomes during COVID‐19. Moreover, the presence of families' resilience is shown because a high proportion of parents reported having support with children during the pandemic; however, the literature consistently found that social support is predictive and a marker of resilience (Kapp & Brown, 2011). Given that family resilience had been shown to be associated with positive outcomes in families with autistic children (Ekas & Rafferty, 2019; Luthar et al., 2000; Ruiz‐Robledillo et al., 2014), and children in general (Bitsika et al., 2013; Ilias et al., 2018), future research should investigate if family resilience had a protective impact on children's outcomes during the COVID‐19 pandemic and uncover the underlying mechanisms.

Evidence of resilience in our findings is also demonstrated in Table 6 because adverse factors had not been found to be associated with average PC scores, however, these were statistically significant predictors of FQoL. Specifically, results show that even though COVID‐19 imposed restrictions to parents' healthcare access were found to be negatively associated with average FQoL scores, these factors were not associated with scores which assessed PC relationships during the pandemic. This evidence suggests positive parenting and the presence of a possible buffering effect, which should be further investigated in future research. In particular, our study suggests that parental resilience might have the potential to buffer the transmission of COVID‐19's negative shocks to PC outcomes. This is consistent with previous research which has shown that sensitive parenting has the ability to buffer the effects of COVID‐19 environments on PC outcomes, as well as, on child psychopathology. (DePasquale & Gunnar, 2020; Feldman, 2020; Vakrat et al., 2018).

While this study focused on three neurogenetic disorders, PMD, RTT, and *SYNGAP1*‐ID, its results are likely generalizable to other neurogenetic disorders with high comorbidity of autism such as Fragile X syndrome. Although FQoL was not specifically measured before and during the COVID‐19 pandemic, one report did suggest that mothers of children with Fragile X syndrome maintained self‐efficacy in caring for their children's behavioral problems during a COVID‐19 related lockdown (Di Giorgio et al., 2021). This again demonstrates the resilience of families with children with syndromic autism in the face limitations imposed by the pandemic and suggests this finding might be more generalizable to idiopathic ID and autism.

Overall, our results provided evidence which might reconcile the contrasting findings around the impact of the pandemic on parents and their relationship with their autistic children (Ameis et al., 2020; Amorim et al., 2020; Asbury et al., 2021; Fontanesi et al., 2020; Fridell et al., 2022; Greenway & Eaton‐Thomas, 2020; Lugo‐Marín et al., 2021; Mumbardó‐Adam et al., 2021; Narzisi, 2020; Pellicano et al., 2021; Tsibidaki, 2021). Thus, while parents with autistic children were exposed to vulnerable COVID‐19 environments, the evidence in our study suggests that those negative shocks were not transmitted to PC outcomes. Furthermore, our findings are consistent with previous research which found that families also benefited from the extra time with their children during COVID‐19 to achieve positive parental–child outcomes (Fontanesi et al., 2020; Fridell et al., 2022; Greenway & Eaton‐Thomas, 2020; Mumbardó‐Adam et al., 2021). This study also echoes the earlier research which found that during crises families may experience resilience and display the ability to handle persistent challenges as well as to thrive in the face of adversity (Calhoun & Tedeschi, 2014; Masten, 2016; Masten & Narayan, 2012).

### Strengths and limitations

The major strength of this study is the longitudinal design which used identical and validated FQoL measures primarily collected during 2018 and the first year of the COVID‐19 pandemic on families with syndromic autistic children. Another major strength of the study is that it collected rich and novel data during COVID‐19 which ascertained exposure‐outcome relationship consistently over and across genetic disorders that strongly predispose to syndromic ASD and ID. This study collected novel data on PC activities and interactions during the COVID‐19 pandemic across different syndromic autism disorders which is also a strength.

The study's main limitation is that participating families in the prepandemic study are not necessarily identical to families with the same conditions that participated in the COVID‐19 study, even though participants in both studies were recruited using identical methods and sample frames. Thus, reported estimates do not necessarily represent causal relationships. Another limitation is that socioeconomic variables were not collected for the prepandemic study and that the participating parents in 2018 and during the pandemic surveys are not necessarily the same. Thus, observed differences in FQoL scores before and during the first year of the pandemic were not adjusted for the socioeconomic factors. However, the studies before and during the pandemic recruited participants using identical methods and patients' registries within a relatively short time‐frame. This suggests that there might be a significant overlap of participating families in both studies. However, the evidence in Table 1 of this study and Appendix B in previously published study (Bolbocean et al., 2021) shows that PMD children age and sex variables collected during COVID‐19 closely match with the demographics of PMD registry in 2018 utilized to recruit participants for our study. Moreover, we randomly matched on clinical diagnosis families with PMD children before and during the first year of the pandemic and our results were consistent with comparison of FQoL scores within the combined sample. Similarly, *SYNGAP1*‐ID registry reported during the prepandemic data collection (Bolbocean et al., 2021) similar distribution of the socio‐economic and demographic covariates. Finally, RTT mostly impacts girls, thus before and during the pandemic observations for RTT implicitly adjusted for a child's sex.

The participating families were registered with charities which suggest that they may be well connected and possibly followed by an informed team of health care providers. Thus, it is possible that the families who did participate in the study were more stressed and they would rate their FQoL differently which might limit the generalizability of our findings. The generalizability of our results might also be limited because the participating families were relatively homogeneous with respect to the socioeconomic gradient of health. In addition, P‐C ad‐hoc index was not a validated measure of parental–child interactions. Finally, due to the nature of data collection process we were not able to confirm the diagnosis in participant families.

## CONCLUSIONS

Despite challenges imposed by the COVID‐19 outbreak, our study found that the quality of life reported by participating families during the first year of COVID‐19 appears to be similar to ratings from a prepandemic study with families with the same conditions, some of whom may have been in the COVID‐19 study. Overall, parents of children in our sample generally displayed a stable functioning trajectory as measured by FQoL instrument. Quality of life outcomes for families who completed surveys before and after the commencement of school activities during September 2020 did not appear to differ. The COVID‐19 pandemic was found to be positively associated with PC bonding, relational satisfaction, and increased emotional connection between parents and their children. Our findings provide evidence of family resilience which might explain positive PC interactions during COVID‐19. Exploring mechanisms which explain how families with autistic and ID children confronted, managed disruptive experiences, and buffered COVID‐19 induced stress is a fruitful direction for future research.

## ETHICS STATEMENT

All study procedures were prospectively reviewed and approved by the institutional review board of the Baylor College of Medicine Houston, TX US.

## Supporting information


**APPENDIX S1:** Supporting Information

## Data Availability

The data that support the findings of this study are available on request from the corresponding author. The data are not publicly available due to privacy or ethical restrictions.

## Footnotes

^1^
Additional details regarding the disorders considered can be found in Appendix A.

^2^
We assessed the agreement between FQoL and P‐C measures using intraclass correlation coefficient (ICC). An ICC less than 0.75 is equivalent to moderate agreement, and greater than 0.75 indicates good agreement (Burke et al., 2017; Weir, 2005).

^3^
We tested the hypothesis that PMD caregivers before and after the cutoff date were different in terms of sociodemographic covariates. A linear probability model was designed to predict before or after cutoff date participation status as a function of the child's age, caregiver's age, sex, race, family income, and primary language spoken at home. The evidence showed that the sociodemographic covariates were not significant predictors of participation status before or after cutoff date.

^4^
The results showed a relatively low level of agreement between FQoL and P‐C scores as the ICC in our data between measures was 0.21. This suggests that the FQoL and P‐C instruments utilized in our study primarily measured different constructs even though both instruments consisted of resilience related constructs.
