# Supplementary material for: Resilience, and positive parenting in parents of children with syndromic autism and intellectual disability. Evidence from the impact of the COVID‐19 pandemic on family's quality of life and parent–child relationships
Source: Autism Res. 2022 Oct 4;15(12):2381–98. doi: 10.1002/aur.2825 (PMC10092377; doi:10.1002/aur.2825)
Supplement: Supplementary file 1 — APPENDIX S1: Supporting Information [file AUR-15-2381-s001.pdf]

A Additional Details related to Diagnoses Considered

Phelan-McDermid syndrome is characterized by an early global developmental delay and is the most frequent reason affected children are referred for neurodevelopmental and genetic evaluation. PMD typically progresses to intellectual disability in nearly 100% of affected individuals (De Rubeis et al., 2018). The most severely impacted developmental domain is language, and this, in combination with social behavior deficits as well as sensory processing abnormalities, frequently leads to a diagnosis of autism (De Rubeis et al., 2018). Approximately 35% of individuals with PMD also develop epilepsy (Phelan et al., 2018, Oberman et al., 2015, Holder Jr and Quach, 2016). The causative genetic abnormality is haploinsufficiency of the SH3 and Multiple Ankyrin Repeats Domains 3 (*SHANK3*) gene (De Rubeis et al., 2018).

Individuals with *SYNGAP1*-ID similarly typically present with a global developmental delay that progresses to a diagnosis of intellectual disability (Holder Jr et al., 2019). In contrast to PMD, over 90% of individuals affected by *SYNGAP1*-ID develop epilepsy (Holder Jr et al., 2019, Jimenez-Gomez et al., 2019). Individuals with SYNGAP1-ID are typically first referred for specialty medical care either for developmental delay or epilepsy. In addition to global developmental delay and epilepsy, children with *SYNGAP1*-ID frequently also have social and sensory processing deficits (Lyons-Warren et al., 2022) which in combination with severe abnormalities in language development, leads to a diagnosis of autism. Haploinsufficiency of the Synaptic Ras GTPase activating protein 1 *SYNGAP1* is the genetic etiology of this disorder (Holder Jr et al., 2019).

For Rett syndrome, early development of affected girls can be normal or slightly delayed with a sudden regression of developmental skills at 1-4 years of age (Neul, 2012). The regression includes loss of both social skills, as well as, language again leading to an autism diagnosis as well as moderate to severe intellectual disability. Uniquely for RTT, prominent loss of purposeful hand use is a pathognomonic feature. In rare cases, boys can be affected with RTT but are typically more severely affected. RTT is most commonly due to Methyl CpG Binding Protein 2 (*MECP2*) loss of function mutations. *MECP2* is located on the X chromosome and primarily impacts girls due to random X-inactivation (Sandweiss et al., 2020).

B Additional Details related to Pre and During FQoL Outcomes

Table B.1: Answers to FQOL Pre and During COVID-19 Pandemic

	Pre-COVID-19	During COVID-19	Total	p-value
N (%)	391 (56.6)	230 (43.4)	621 (100.0)	
My family enjoys spending time together, n (%)				
Dissatisfied	40 (10.2)	12 (8.9)	52 (9.9)	
Neither	36 (9.2)	13 (9.6)	49 (9.3)	
Satisfied	170 (43.5)	54 (40.0)	224 (42.6)	
Very Dissatisfied	17 (4.3)	5 (3.7)	22 (4.2)	
Very Satisfied	128 (32.7)	51 (37.8)	179 (34.0)	0.86
My family members talk openly with each other, n (%)				
Dissatisfied	40 (10.3)	14 (10.4)	54 (10.3)	
Neither	51 (13.2)	18 (13.3)	69 (13.2)	
Satisfied	174 (45.0)	68 (50.4)	242 (46.4)	
Very Dissatisfied	20 (5.2)	3 (2.2)	23 (4.4)	
Very Satisfied	102 (26.4)	32 (23.7)	134 (25.7)	0.58
Our family solves problems together, n (%)				
Dissatisfied	41 (10.5)	10 (7.5)	51 (9.7)	
Neither	48 (12.3)	23 (17.2)	71 (13.5)	
Satisfied	189 (48.5)	66 (49.3)	255 (48.7)	
Very Dissatisfied	18 (4.6)	3 (2.2)	21 (4.0)	
Very Satisfied	94 (24.1)	32 (23.9)	126 (24.0)	0.39
My family members support each other to accomplish goals, n (%)				
Dissatisfied	37 (9.5)	4 (3.0)	41 (7.8)	
Neither	51 (13.0)	18 (13.3)	69 (13.1)	
Satisfied	198 (50.6)	76 (56.3)	274 (52.1)	
Very Dissatisfied	11 (2.8)	2 (1.5)	13 (2.5)	
Very Satisfied	94 (24.0)	35 (25.9)	129 (24.5)	0.14
My family members show that they love and care for each other, n (%)				
Dissatisfied	19 (4.9)	1 (0.7)	20 (3.8)	
Neither	17 (4.3)	13 (9.7)	30 (5.7)	
Satisfied	179 (45.8)	58 (43.3)	237 (45.1)	
Very Dissatisfied	7 (1.8)	1 (0.7)	8 (1.5)	
Very Satisfied	169 (43.2)	61 (45.5)	230 (43.8)	0.03
My family is able to handle life's ups and downs, n (%)				
Dissatisfied	46 (11.8)	10 (7.4)	56 (10.6)	
Neither	67 (17.1)	18 (13.3)	85 (16.2)	
Satisfied	194 (49.6)	74 (54.8)	268 (51.0)	
Very Dissatisfied	8 (2.0)	1 (0.7)	9 (1.7)	
Very Satisfied	76 (19.4)	32 (23.7)	108 (20.5)	0.28
My family members help the children learn to be independent, n (%)				
Dissatisfied	65 (16.8)	16 (11.9)	81 (15.5)	
Neither	74 (19.1)	31 (23.0)	105 (20.1)	
Satisfied	172 (44.4)	59 (43.7)	231 (44.3)	
Very Dissatisfied	15 (3.9)	5 (3.7)	20 (3.8)	
Very Satisfied	61 (15.8)	24 (17.8)	85 (16.3)	0.63
My family members help the children with schoolwork and activities, n (%)				
Dissatisfied	66 (17.0)	26 (19.3)	92 (17.6)	
Neither	86 (22.2)	39 (28.9)	125 (23.9)	
Satisfied	140 (36.1)	41 (30.4)	181 (34.6)	
Very Dissatisfied	49 (12.6)	15 (11.1)	64 (12.2)	
Very Satisfied	47 (12.1)	14 (10.4)	61 (11.7)	0.47
My family members teach the children how to get along with each other, n (%)				
Dissatisfied	30 (7.8)	8 (6.1)	38 (7.3)	
Neither	66 (17.1)	29 (22.0)	95 (18.3)	
Satisfied	185 (47.8)	65 (49.2)	250 (48.2)	
Very Dissatisfied	13 (3.4)	2 (1.5)	15 (2.9)	
Very Satisfied	93 (24.0)	28 (21.2)	121 (23.3)	0.52
Adults in our family teach the children to make good decisions, n (%)				
Dissatisfied	20 (5.2)	5 (3.7)	25 (4.8)	
Neither	51 (13.2)	16 (11.9)	67 (12.9)	
Satisfied	198 (51.4)	71 (53.0)	269 (51.8)	
Very Dissatisfied	7 (1.8)	3 (2.2)	10 (1.9)	
Very Satisfied	109 (28.3)	39 (29.1)	148 (28.5)	0.95
Adults in my family know other people in the children's lives (friends, teachers, etc.), n (%)				
Dissatisfied	43 (11.1)	10 (7.4)	53 (10.1)	
Neither	56 (14.4)	15 (11.1)	71 (13.6)	
Satisfied	172 (44.3)	67 (49.6)	239 (45.7)	
Very Dissatisfied	11 (2.8)	4 (3.0)	15 (2.9)	
Very Satisfied	106 (27.3)	39 (28.9)	145 (27.7)	0.58
Adults in my family have time to take care of the individual needs of every child, n (%)				
Dissatisfied	74 (19.1)	19 (14.1)	93 (17.8)	
Neither	58 (14.9)	24 (17.8)	82 (15.7)	
Satisfied	164 (42.3)	60 (44.4)	224 (42.8)	
Very Dissatisfied	24 (6.2)	7 (5.2)	31 (5.9)	
Very Satisfied	68 (17.5)	25 (18.5)	93 (17.8)	0.69
My family has the support we need to relieve stress, n (%)				
Dissatisfied	115 (29.6)	38 (28.1)	153 (29.2)	
Neither	60 (15.4)	20 (14.8)	80 (15.3)	
Satisfied	118 (30.3)	45 (33.3)	163 (31.1)	
Very Dissatisfied	64 (16.5)	17 (12.6)	81 (15.5)	
Very Satisfied	32 (8.2)	15 (11.1)	47 (9.0)	0.68
My family members have friends or others who provide support, n (%)				
Dissatisfied	86 (22.1)	28 (20.7)	114 (21.7)	
Neither	69 (17.7)	22 (16.3)	91 (17.3)	
Satisfied	147 (37.7)	56 (41.5)	203 (38.7)	
Very Dissatisfied	52 (13.3)	13 (9.6)	65 (12.4)	
Very Satisfied	36 (9.2)	16 (11.9)	52 (9.9)	0.67
My family members have some time to pursue our own interests, n (%)				
Dissatisfied	118 (30.5)	26 (19.4)	144 (27.6)	
Neither	76 (19.6)	21 (15.7)	97 (18.6)	
Satisfied	108 (27.9)	57 (42.5)	165 (31.7)	
Very Dissatisfied	43 (11.1)	8 (6.0)	51 (9.8)	
Very Satisfied	42 (10.9)	22 (16.4)	64 (12.3)	0.00
My family has outside help available to us to take care of special needs of all family members, n (%)				
Dissatisfied	98 (25.1)	28 (20.7)	126 (24.0)	
Neither	69 (17.7)	30 (22.2)	99 (18.9)	
Satisfied	97 (24.9)	33 (24.4)	130 (24.8)	
Very Dissatisfied	78 (20.0)	25 (18.5)	103 (19.6)	
Very Satisfied	48 (12.3)	19 (14.1)	67 (12.8)	0.69
My family gets medical care when needed, n (%)				
Dissatisfied	15 (3.9)	8 (5.9)	23 (4.4)	
Neither	27 (6.9)	9 (6.7)	36 (6.9)	
Satisfied	153 (39.3)	65 (48.1)	218 (41.6)	
Very Dissatisfied	9 (2.3)	2 (1.5)	11 (2.1)	
Very Satisfied	185 (47.6)	51 (37.8)	236 (45.0)	0.26
My family gets dental care when needed, n (%)				
Dissatisfied	32 (8.2)	11 (8.1)	43 (8.2)	
Neither	18 (4.6)	11 (8.1)	29 (5.5)	
Satisfied	151 (38.7)	51 (37.8)	202 (38.5)	
Very Dissatisfied	14 (3.6)	4 (3.0)	18 (3.4)	
Very Satisfied	175 (44.9)	58 (43.0)	233 (44.4)	0.65
My family members have transportation to get to the places they need to be, n (%)				
Dissatisfied	37 (9.5)	6 (4.5)	43 (8.2)	
Neither	32 (8.2)	10 (7.5)	42 (8.0)	
Satisfied	151 (38.7)	60 (44.8)	211 (40.3)	
Very Dissatisfied	20 (5.1)	3 (2.2)	23 (4.4)	
Very Satisfied	150 (38.5)	55 (41.0)	205 (39.1)	0.19
My family has a way to take care of our expenses, n (%)				
Dissatisfied	40 (10.3)	7 (5.2)	47 (9.0)	
Neither	55 (14.2)	14 (10.4)	69 (13.2)	
Satisfied	156 (40.2)	62 (46.3)	218 (41.8)	
Very Dissatisfied	24 (6.2)	5 (3.7)	29 (5.6)	
Very Satisfied	113 (29.1)	46 (34.3)	159 (30.5)	0.15
My family feels safe at home, work, school, and in our neighborhood, n (%)				
Dissatisfied	13 (3.3)	3 (2.2)	16 (3.0)	
Neither	13 (3.3)	7 (5.2)	20 (3.8)	
Satisfied	161 (41.3)	63 (46.7)	224 (42.7)	
Very Dissatisfied	4 (1.0)	2 (1.5)	6 (1.1)	
Very Satisfied	199 (51.0)	60 (44.4)	259 (49.3)	0.55
My family member with a disability has support to accomplish goals at school or at workplace, n (%)				
Dissatisfied	40 (10.4)	15 (11.2)	55 (10.6)	
Neither	47 (12.2)	18 (13.4)	65 (12.5)	
Satisfied	179 (46.5)	56 (41.8)	235 (45.3)	
Very Dissatisfied	17 (4.4)	8 (6.0)	25 (4.8)	
Very Satisfied	102 (26.5)	37 (27.6)	139 (26.8)	0.88
My family member with a disability has support to accomplish goals at home, n (%)				
Dissatisfied	40 (10.3)	11 (8.1)	51 (9.8)	
Neither	54 (13.9)	20 (14.8)	74 (14.1)	
Satisfied	181 (46.6)	55 (40.7)	236 (45.1)	
Very Dissatisfied	8 (2.1)	6 (4.4)	14 (2.7)	
Very Satisfied	105 (27.1)	43 (31.9)	148 (28.3)	0.37
My family member with a disability has support to make friends, n (%)				
Dissatisfied	76 (19.5)	32 (23.9)	108 (20.7)	
Neither	83 (21.3)	36 (26.9)	119 (22.8)	
Satisfied	135 (34.7)	41 (30.6)	176 (33.7)	
Very Dissatisfied	39 (10.0)	8 (6.0)	47 (9.0)	
Very Satisfied	56 (14.4)	17 (12.7)	73 (14.0)	0.31
My family has good relationships with the service providers who provide services and support to our family members with a disability, n (%)				
Dissatisfied	35 (9.0)	13 (9.6)	48 (9.2)	
Neither	60 (15.4)	14 (10.4)	74 (14.1)	
Satisfied	162 (41.6)	58 (43.0)	220 (42.0)	
Very Dissatisfied	8 (2.1)	7 (5.2)	15 (2.9)	
Very Satisfied	124 (31.9)	43 (31.9)	167 (31.9)	0.26

Notes: p-value is based on Fisher's exact test

Table B.2: FQOL Scores Pre and During COVID-19 Pandemic

	**Pre-COVID-19**	**During COVID-19**	**Total**	***p*-value**
n (%)	391 (56.6)	230 (43.4)	621 (100.0)	
My family enjoys spending time together, median (IQI)	3.00 (3.00; 5.00)	3.00 (3.00; 5.00)	3.00 (3.00; 5.00)	0.41
My family members talk openly with each other, median (IQI)	3.00 (3.00; 5.00)	3.00 (3.00; 4.00)	3.00 (3.00; 5.00)	0.51
Our family solves problems together, median (IQI)	3.00 (3.00; 4.00)	3.00 (2.75; 4.00)	3.00 (3.00; 4.00)	0.75
My family members support each other to accomplish goals, median (IQI)	3.00 (3.00; 4.00)	3.00 (3.00; 5.00)	3.00 (3.00; 4.00)	0.28
My family members show that they love and care for each other, median (IQI)	3.00 (3.00; 5.00)	3.00 (3.00; 5.00)	3.00 (3.00; 5.00)	0.81
My family is able to handle life's ups and downs, median (IQI)	3.00 (2.00; 3.00)	3.00 (3.00; 3.00)	3.00 (2.00; 3.00)	0.11
My family members help the children learn to be independent, median (IQI)	3.00 (2.00; 3.00)	3.00 (2.00; 3.00)	3.00 (2.00; 3.00)	0.51
My family members help the children with schoolwork and activities, median (IQI)	3.00 (2.00; 3.00)	3.00 (2.00; 3.00)	3.00 (2.00; 3.00)	0.16
My family members teach the children how to get along with each other, median (IQI)	3.00 (3.00; 4.00)	3.00 (2.00; 3.00)	3.00 (2.00; 4.00)	0.41
Adults in our family teach the children to make good decisions, median (IQI)	3.00 (3.00; 5.00)	3.00 (3.00; 5.00)	3.00 (3.00; 5.00)	0.61
Adults in my family know other people in the children's lives (friends, teachers, etc.), median (IQI)	3.00 (2.00; 5.00)	3.00 (3.00; 5.00)	3.00 (3.00; 5.00)	0.27
Adults in my family have time to take care of the individual needs of every child, median (IQI)	3.00 (2.00; 3.00)	3.00 (2.00; 3.00)	3.00 (2.00; 3.00)	0.58
My family has the support we need to relieve stress, median (IQI)	3.00 (1.00; 3.00)	3.00 (1.00; 3.00)	3.00 (1.00; 3.00)	0.75
My family members have friends or others who provide support, median (IQI)	3.00 (2.00; 3.00)	3.00 (2.00; 3.00)	3.00 (2.00; 3.00)	0.72
My family members have some time to pursue our own interests, median (IQI)	2.00 (1.00; 3.00)	3.00 (2.00; 3.00)	3.00 (1.00; 3.00)	0.01
My family has outside help available to us to take care of special needs of all family members, median (IQI)	3.00 (1.00; 4.00)	3.00 (2.00; 4.00)	3.00 (2.00; 4.00)	0.66
My family gets medical care when needed, median (IQI)	3.00 (3.00; 5.00)	3.00 (3.00; 5.00)	3.00 (3.00; 5.00)	0.07
My family gets dental care when needed, median (IQI)	3.00 (3.00; 5.00)	3.00 (3.00; 5.00)	3.00 (3.00; 5.00)	0.54
My family members have transportation to get to the places they need to be, median (IQI)	3.00 (3.00; 5.00)	3.00 (3.00; 5.00)	3.00 (3.00; 5.00)	0.43
My family has a way to take care of our expenses, median (IQI)	3.00 (3.00; 5.00)	3.00 (3.00; 5.00)	3.00 (3.00; 5.00)	0.11
My family feels safe at home, work, school, and in our neighborhood, median (IQI)	5.00 (3.00; 5.00)	3.00 (3.00; 5.00)	4.00 (3.00; 5.00)	0.28
My family member with a disability has support to accomplish goals at school or at workplace, median (IQI)	3.00 (3.00; 5.00)	3.00 (2.75; 5.00)	3.00 (3.00; 5.00)	0.95
My family member with a disability has support to accomplish goals at home, median (IQI)	3.00 (3.00; 5.00)	3.00 (3.00; 5.00)	3.00 (3.00; 5.00)	0.28
My family member with a disability has support to make friends, median (IQI)	3.00 (2.00; 3.00)	2.00 (2.00; 3.00)	3.00 (2.00; 3.00)	0.08
My family has good relationships with the service providers, median (IQI)	3.00 (3.00; 5.00)	3.00 (3.00; 5.00)	3.00 (3.00; 5.00)	0.52

Notes: IQI - interquartile interval. *p*-value is based on Kruskal Wallis test for medians.
